# Arginine:Glycine Amidinotransferase Is Essential for Creatine Supply in Mice During Chronic Hypoxia

**DOI:** 10.3389/fphys.2021.703069

**Published:** 2021-08-18

**Authors:** Juliane Hannemann, Kathrin Cordts, Anika Seniuk, Chi-un Choe, Lena Schmidt-Hutten, Jorge Duque Escobar, Florian Weinberger, Rainer Böger, Edzard Schwedhelm

**Affiliations:** ^1^Institute of Clinical Pharmacology and Toxicology, University Medical Center Hamburg-Eppendorf, Hamburg, Germany; ^2^Institute DECIPHER, German-Chilean Institute for Research on Pulmonary Hypoxia and Its Health Sequelae, Hamburg, Germany; ^3^German Centre for Cardiovascular Research (DZHK), Partner Site Hamburg/Kiel/Lübeck, Hamburg, Germany; ^4^Institute of Cellular and Integrative Physiology, University Medical Center Hamburg, Hamburg, Germany; ^5^Department of Neurology, University Medical Center Hamburg-Eppendorf, Hamburg, Germany; ^6^Department of Cardiology, University Heart and Vascular Center, Hamburg, Germany; ^7^Insitute of Experimental Pharmacology and Toxicology, University Medical Center Hamburg-Eppendorf, Hamburg, Germany

**Keywords:** creatine, homoarginine, hypoxia, L-arginine:glycine amidinotransferase, pulmonary hypertension

## Abstract

**Objective:** Chronic hypoxia induces pulmonary and cardiovascular pathologies, including pulmonary hypertension (PH). L-arginine:glycine amidinotransferase (AGAT) is essential for homoarginine (hArg) and guanidinoacetate synthesis, the latter being converted to creatine by guanidinoacetate methyltransferase. Low hArg concentrations are associated with cardiovascular morbidity and predict mortality in patients with PH. We therefore aimed to investigate the survival and cardiac outcome of AGAT knockout (*Agat*^−/−^) mice under hypoxia and a possible rescue of the phenotype.

**Methods:***Agat*^−/−^ mice and wild-type (WT) littermates were subjected to normoxia or normobaric hypoxia (10% oxygen) for 4 weeks. A subgroup of *Agat*^−/−^ mice was supplemented with 1% creatine from weaning. Survival, hematocrit, blood lactate and glucose, heart weight-to-tibia length (HW/TL) ratio, hArg plasma concentration, and *Agat* and *Gamt* expression in lung, liver, and kidneys were evaluated.

**Results:** After 6 h of hypoxia, blood lactate was lower in *Agat*^−/−^-mice as compared to normoxia (*p* < 0.001). *Agat*^−/−^ mice died within 2 days of hypoxia, whereas *Agat*^−/−^ mice supplemented with creatine and WT mice survived until the end of the study. In WT mice, hematocrit (74 ± 4 vs. 55 ± 2%, mean ± SD, *p* < 0.001) and HW/TL (9.9 ± 1.3 vs. 7.3 ± 0.7 mg/mm, *p* < 0.01) were higher in hypoxia, while hArg plasma concentration (0.25 ± 0.06 vs. 0.38 ± 0.12 μmol/L, *p* < 0.01) was lower. *Agat* and *Gamt* expressions were differentially downregulated by hypoxia in lung, liver, and kidneys.

**Conclusion:***Agat* and *Gamt* are downregulated in hypoxia. *Agat^−/−^* mice are nonviable in hypoxia. Creatine rescues the lethal phenotype, but it does not reduce right ventricular hypertrophy of *Agat^−/−^* mice in hypoxia.

## Introduction

In patients with newly diagnosed, treatment-naïve pulmonary hypertension (PH), low homoarginine (hArg) plasma concentration was identified as an independent predictor of morbidity and mortality (Atzler et al., 2016). PH is a rare disease with a poor prognosis; it is based on an increase in vascular resistance, which leads to elevated blood pressure in the pulmonary circulation. The increased afterload leads to an increased stress on the right ventricle, causing right ventricular hypertrophy. Eventually, heart failure evolves, which is a major cause of death in PH patients (Giordano, 2005).

The detailed pathophysiological mechanisms leading to idiopathic pulmonary hypertension are not yet fully understood. Pulmonary vasoconstriction is one of the compensatory mechanisms that help to maintain sufficient oxygen supply in focal pulmonary hypoxia; however, excessive and global pulmonary vasoconstriction in global hypoxia may lead to pulmonary hypertension and fatal outcome (Hannemann et al., 2020b). Increased vasoconstriction of the pulmonary vessels is assumed to play an important role, due to an increased release of vasoconstrictor prostaglandins and endothelin-1, and a reduced release of the vasodilator nitric oxide (NO; Lourenco et al., 2012; Giannakoulas et al., 2014). Accumulation of endogenous inhibitors of NO synthesis contributed to this dysregulation, and we and others have shown that chronic hypoxia induces pulmonary and cardiovascular phenotypes that are similar to PH (Hannemann et al., 2020a; Smith et al., 2020); therefore, chronic hypoxia was used as an experimental model in this study.

hArg is a non-proteinogenic amino acid that is synthesized by L-arginine:glycine amidinotransferase (AGAT, Kayacelebi et al., 2015). The same enzyme catalyzes the formation of guanidinoacetic acid, which is further metabolized to creatine by guanidinoacetate methyltransferase (GAMT, Kan et al., 2004). Accordingly, *Agat*-deficient mice (*Agat*^−/−^ mice) are characterized by low circulating hArg and creatine concentrations (Choe et al., 2013a; Nabuurs et al., 2013). hArg is a weak substrate of NO synthase (NOS) and an inhibitor of arginase (Hrabák et al., 1994; Moali et al., 1998). Phosphorylated creatine, on the other hand, plays a key role in energy metabolism and acts as a rapidly available energy buffer due to its capacity to recycle adenosine triphosphate (ATP, Lygate et al., 2013; Stockebrand et al., 2018).

Our group has previously shown that *Agat*^−/−^ mice develop significantly larger stroke areas in a temporary middle cerebral artery occlusion model as compared to WT littermates. Stroke size was significantly reduced to WT size in *Agat*^−/−^ mice supplemented with hArg, but only slightly in creatine-supplemented animals (Choe et al., 2013a). Likewise, Faller and co-workers have shown that impaired cardiac contractile function in *Agat*^−/−^ mice is rescued by hArg, but not creatine (Faller et al., 2018). By contrast, Laasmaa et al. showed that impaired calcium handling by isolated cardiomyocytes from *Agat*^−/−^ mice is normalized by creatine supplementation when creatine is supplemented from weaning (Laasmaa et al., 2021). Taken together, these data suggest that hArg and creatine play differential roles in mediating the pathophysiological phenotypes of *Agat*^−/−^ mice, which are not yet fully understood.

Chronic hypoxia is a condition that is associated with a global dysbalance of energy metabolism. We therefore hypothesized that *Agat*^−/−^ mice may be specifically vulnerable in chronic hypoxia and that creatine or hArg supplementation may help to maintain a healthy phenotype.

## Materials and Methods

### Animals and Study Design

*Agat*^−/−^ mice used in the present study were described previously and have a life expectancy similar to WT littermates (Choe et al., 2013a,b; Nabuurs et al., 2013). Female mice were kept at 20 to 24°C and a 12:12 light–dark cycle with free access to chow and water. Agat^−/−^ mice and WT littermates aged 3–4 months were placed in normobaric hypoxia (10% O_2_) or normoxia (21% O_2_) for 4 weeks (8–9 animals per group, Supplementary Figure 1). A separate group of *Agat^−/−^* mice was supplemented with 1% creatine from weaning in standard chow (Ssniff, Soest, Germany) and placed in normoxia or hypoxia, respectively, together with WT littermates in hypoxia (8–9 animals per group). For each group separately, mice from the same litter were kept in one cage. Throughout the study period, all animals were monitored daily for survival and chow was changed from dry to moistened starting 4 to 7 days prior to hypoxia for *Agat*^*−/*−^ mice. For the first hours of hypoxia, mice were housed on external heating mates. After 6 h of hypoxia, a blood sample was collected by puncture; surviving animals were killed in anesthesia after 4 weeks of hypoxia or normoxia. Blood and tissues were collected and processed for analysis as previously described (Hannemann et al., 2020a). This study was approved by the Animal Care and Use Committee of the Freie und Hansestadt Hamburg (Approval no. 40/16). The use of animals was consistent with the Guide for the Care and Use of Laboratory Animals published by the US National Institutes of Health (NIH publication No. 85-23, revised 1996).

### Analysis of Hematocrit, Glucose, Lactate, Cardiac Troponin I, Hypertrophy, and Echocardiography

Hematocrit, glucose, and lactate were determined after puncture of the *Vena facialis* and subsequent blood analysis with an ABL 90 FLEX (Radiometer, Krefeld, Germany) after 6 h of hypoxia and after 4 weeks of hypoxia or normoxia. Cardiac troponin I (cTnI) was determined by ARCHITECT STAT high-sensitive cTnI immunoassay (i2000SR, Abbott Diagnostics, IL, United States) in serum samples diluted 1:5 with 0.9% NaCl. The epitope of the antigen showed 96% similarity between mice and human. Body weight and wet weights of the right ventricle (RV) and left ventricle (LV), septum, and the tibia length were determined. The Fulton index was calculated by dividing the RV to the sum of LV and septum. Cardiac echography was performed using the Vevo 3100 System (VisualSonics, Toronto, Canada) using standardized procedures (Zacchigna et al., 2021). Mice were anaesthetized with isoflurane (1–2%) and taped to a warming platform in a supine position. B-mode images were obtained using a MX400 transducer. Images were obtained in a parasternal short- and long-axis view, and dimensions of the left ventricle were measured in a short-axis view in diastole and systole. Fractional area shortening (FAS) was calculated as:

FAS (%) =(LVIDd-LVIDs)/LVIDd×100

### Quantification of Homoarginine Plasma Concentration

hArg plasma concentrations were determined from EDTA plasma obtained from cardiac puncture; blood samples were centrifuged at 3,000 × *g* for 3 min at 4°C and stored at −80°C until analysis. hArg concentration was determined by liquid chromatography–tandem mass spectrometry (LC–MS/MS) as previously described (Atzler et al., 2011). Briefly, 25 μl of plasma was diluted with 100 μl of stable isotope-labeled hArg dissolved in methanol. After protein precipitation, hArg was converted to its butyl ester derivative and analyzed by LC–MS/MS (Varian 1200, Agilent Technologies, Santa Clara, CA, United States). Quantification was performed by calculation of peak area ratios and calibration with known concentrations of hArg in dialyzed EDTA plasma (Atzler et al., 2011).

### Analysis of *Agat* and *Gamt* mRNA Expression

RNA preparation, reverse transcription, and quantitative real-time PCR (qRT-PCR) were performed as previously described (Hannemann et al., 2020a). Briefly, total RNA was isolated from snap-frozen, homogenized lung, liver, and renal tissues using Trizol (Thermo Fisher Scientific, Waltham, MA, United States). Genomic DNA was removed by an on-column clean-up procedure using PureLink^™^ RNA Mini Kit in combination with PureLink^™^ DNase (both Thermo Fisher). RNA was reverse transcribed with SuperScript^™^ IV VILO^™^ (Thermo Fisher). qRT-PCR was performed with gene-specific, FAM-labeled Taqman^™^ assays for Agat (Mm00491882_m1) and Gamt (Mm00487473_m1) and a VIC-labeled control assay (Tbp, Mm01277042_m1) for normalization (all Thermo Fisher). For all experiment, technical triplicates were analyzed. Relative gene expression was calculated using the ΔΔCt method (Schmittgen and Livak, 2008).

### Analysis of *Agat* Protein Expression

Preparation of tissue lysates was performed as previously described (Hannemann et al., 2020a). Samples were prepared for SDS-PAGE (kidney, 30 μg total protein; liver, 50 μg total protein per lane) by boiling in beta-mercaptoethanol containing sample buffer; samples were subjected to 10% SDS-PAGE. Subsequently, proteins were transferred onto a 0.2 μm nitrocellulose membrane (GE Healthcare, Munich, Germany). Membranes were incubated with the primary antibody (anti-AGAT, 1:300 in liver, 1:500 in kidney; 12801, Proteintech Chicago, Illinois, United States; anti-β-tubulin: 1:500, Abcam ab6046, Cambridge, United Kingdom) for 1 h at room temperature. Incubation with the secondary antibody (1:10,000; A0545, Merck, Darmstadt, Germany) was done for 1 h at room temperature. Protein was detected using enhanced chemiluminescence detection according to the manufacturer’s protocol (GE Healthcare, Munich, Germany).

### Statistical Analyses

Data are expressed as mean ± standard deviation (SD). A two-sided independent Student’s *t*-test was used to compare two groups. One-way ANOVA with Bonferroni *post-hoc* test was used to compare more than two groups. Chi-square test was used to compare dichotomized variables; Kaplan–Meier curves were generated for survival analysis. *p* < 0.05 was considered statistically significant in all analyses. Statistical analyses were performed by GraphPad Prism 6.0 (La Jolla, CA, United States).

## Results

### Acute Effects of Hypoxia in *Agat*^−/−^ Mice vs. WT Littermates

**Figure 1:**
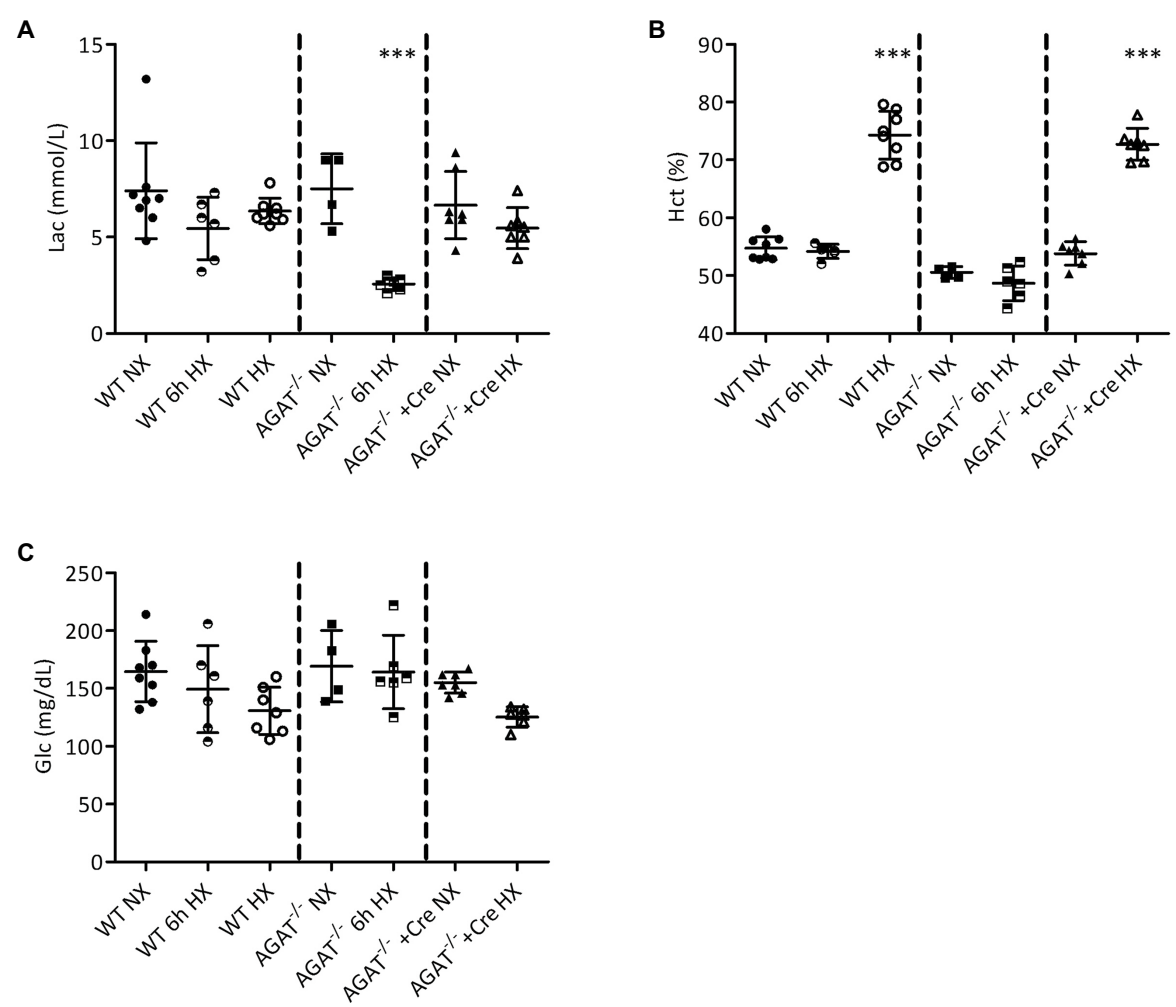
Blood analyses of *Agat^−/−^* mice and wild-type (WT) littermates. **(A–C)** Mean values ± SD from blood gas analyses under normoxia (NX), after 6 h of hypoxia (6 h HX) and after 4 weeks of hypoxia (HX) in unsupplemented and *Agat^−/−^* mice supplemented with 1% creatine from weaning (Cre). **(A)** Lactate (Lac). **(B)** Hematocrit (Hct %). **(C)** Glucose (Glc). *Agat*, arginine:glycine amidinotransferase; Cre, supplemented with 1% creatine. ^***^p < 0.001 vs. NX (ANOVA with Bonferroni *post-hoc* test).

We analyzed the acute effects of hypoxia after 6 h of exposure to 10% oxygen. By comparison with normoxia, there was a significant reduction of blood lactate concentration in *Agat^−/−^* mice (7.5 ± 1.8 vs. 2.6 ± 0.3 mmol/L; *p* < 0.001), while no significant reduction was observed in WT littermates (Figure 1A). No significant difference was observed in hematocrit or glucose after 6 h of hypoxia in *Agat^−/−^* or WT animals (Figures 1B,C).

**Figure 2:**
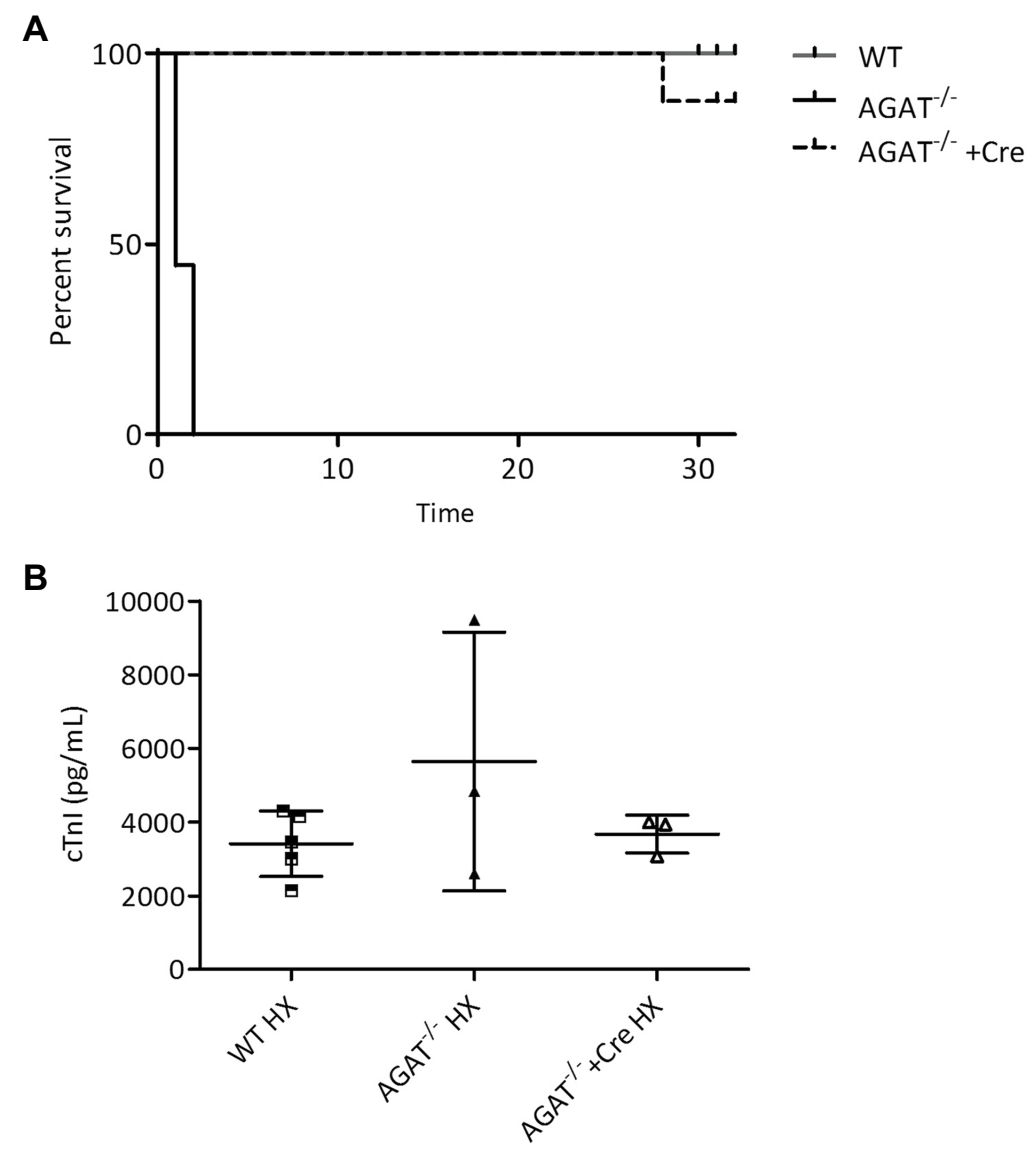
Kaplan–Meier survival analysis and cardiac troponin I (cTnI). **(A,B)** Outcome of *Agat^−/−^* mice with and w/o creatine (Cre) supplementation and WT littermates in hypoxia (HX). **(A)** Kaplan–Meier survival analysis of *Agat^−/−^* mice (*n* = 9), WT littermates (WT, *n* = 17) and *Agat^−/−^* mice supplemented with 1% creatine from weaning (*Agat^−/−^*+Cre, *n* = 8). All mice were kept in hypoxia and followed for up to 32 days. **(B)** cTnI in *Agat^−/−^* mice (*n* = 3, 1–2 days of HX), WT littermates (WT, *n* = 5, 4 weeks of HX) and *Agat^−/−^* mice supplemented with 1% creatine from weaning (*Agat^−/−^*+Cre, *n* = 3, 4 weeks of HX) *Agat*, arginine:glycine amidinotransferase; Cre, supplemented with 1% creatine.

All *Agat^−/−^* mice died within 48 h of hypoxia, whereas all WT littermates survived until the end of the study after 4 weeks of hypoxia (*p* < 0.001 Chi-square test; Figure 2A). The lethal phenotype in hypoxia was completely reversed in *Agat^−/−^* mice supplemented with creatine (Figure 2A). *Agat^−/−^* mice showed a trend of cTnI increase as compared with WT littermates and *Agat^−/−^*-mice supplemented with creatine in hypoxia (Figure 2B).

### Effects of Chronic Hypoxia on *Agat* and *Gamt* Expression and hArg Plasma Concentration in WT Mice

**Figure 3:**
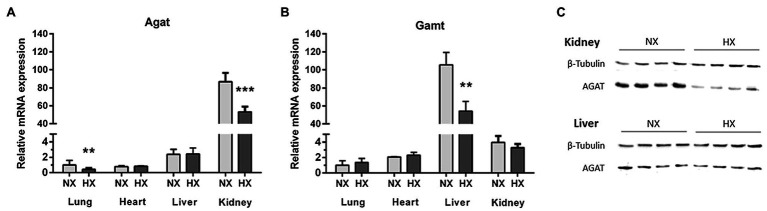
*Agat* and *Gamt* mRNA expression in lungs, liver, heart, and kidneys, and L-arginine:glycine amidinotransferase (AGAT) protein expression in liver and kidneys. mRNA expression of *Agat* **(A)** and *Gamt* **(B)** in normoxia (NX) and hypoxia (HX) as determined by quantitative real-time PCR normalized to Tbp as housekeeping gene. **(C)** Western Blot of AGAT in liver and kidneys as compared to β-tubulin as loading control. ^**^p < 0.01 and ^***^p < 0.001 vs. NX (ANOVA with Bonferroni *post-hoc* test).

*Agat* mRNA was highly expressed in kidneys and to a lower extent in liver, heart, and lungs, while Gamt showed highest gene expression in liver and low gene expression levels in kidneys, heart, and lungs (Figure 3). As compared to normoxia, chronic hypoxia caused a significant reduction of *Agat* mRNA expression in kidneys (*p* = 0.001) and lungs (*p* = 0.005), but not in heart and liver (Figure 3A). Likewise, *Gamt* gene expression in liver was lower in chronic hypoxia (*p* = 0.001), but remained unchanged in kidneys, heart, and lungs (Figure 3B). In line with the gene expression results, AGAT protein is downregulated in the hypoxic kidney. In the liver, where *Agat* expression is rather low, no effect of hypoxia was obvious (Figure 3C). Plasma hArg concentrations were significantly lower in hypoxia as compared to normoxia (0.25 ± 0.06 vs. 0.38 ± 0.12 μmol/L, *p* < 0.01).

### Effects of Creatine Supplementation in *Agat^−/−^* Mice

At the beginning of the intervention, *Agat^−/−^* mice had a lower mean body weight than their WT littermates (13.3 ± 1.3 vs. 19.0 ± 1.7 g, respectively, *p* < 0.001). Agat^−/−^ mice supplemented with creatine from weaning had a body weight similar to WT littermates (18.0 ± 1.4 g, *p* = n.s. vs. WT).

After 4 weeks of hypoxia, hematocrit was significantly elevated in *Agat*^−/−^ mice supplemented with creatine (73 ± 3% vs. 54 ± 2%, *p* < 0.001) and their unsupplemented WT littermates (74 ± 4% vs. 55 ± 2%, *p* < 0.001; Figure 1B) as compared with normoxic littermates. No significant differences in blood lactate and glucose concentrations were observed between mice of both groups exposed to either normoxia or hypoxia (Figures 1A,C).

**Figure 4:**
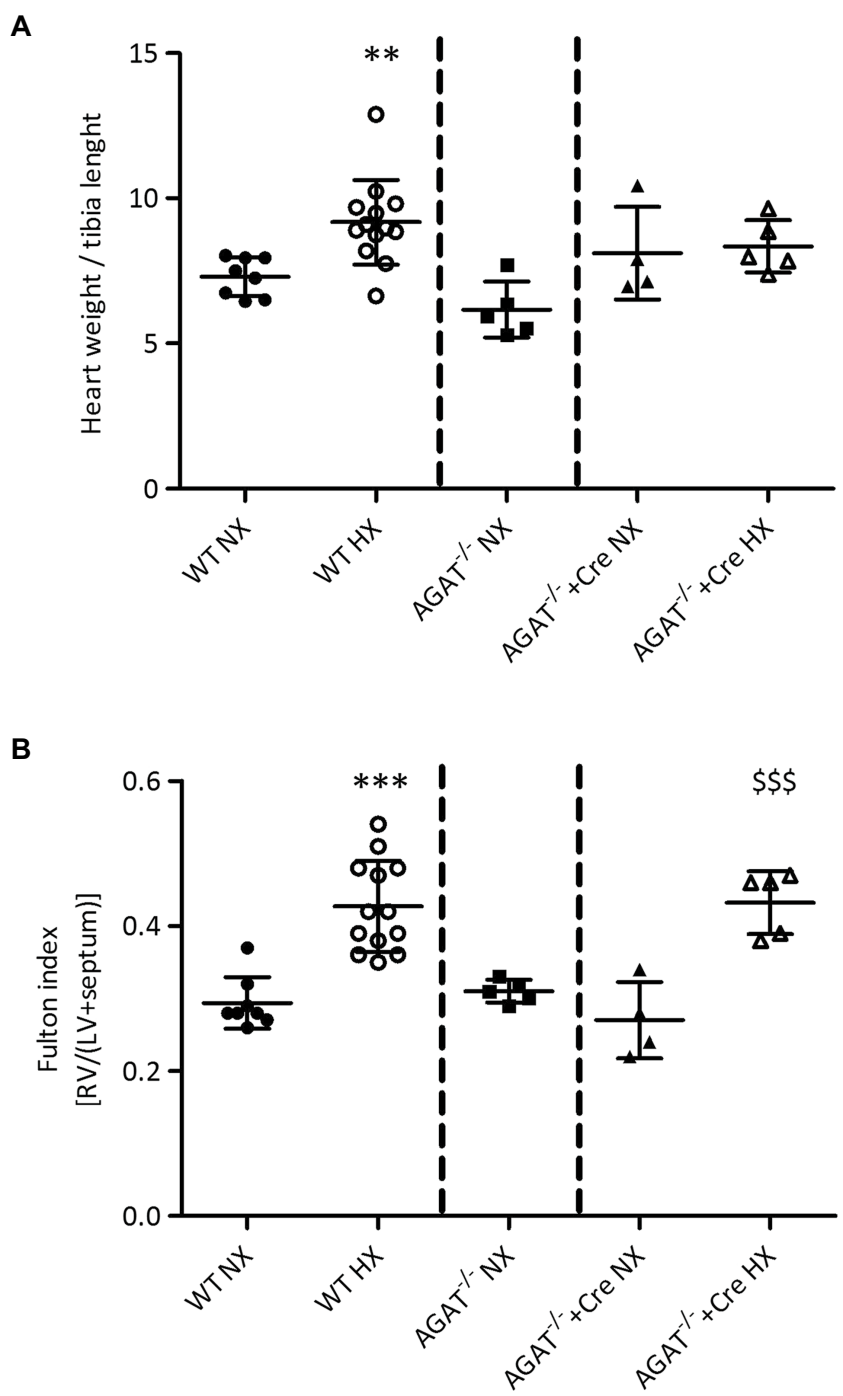
Phenotypes of cardiac hypertrophy of *Agat^−/−^* mice and WT littermates. **(A,B)** Mean values ± SD from hearts from *Agat^−/−^* mice supplemented with 1% creatine and WT littermates (WT) under normoxia (NX) and after 4 weeks of hypoxia (HX). **(A)** Heart weight to tibia length. **(B)** Fulton index {right ventricle (RV) weight/[left ventricle (LV) + septum weight]}. *Agat*, arginine:glycine amidinotransferase; Cre, supplemented with 1% creatine. ^**^p < 0.01 vs. WT NX, ^***^p < 0.001 vs. WT NX, ^$$$^*p* < 0.001 vs. *Agat^−/−^*+Cre NX (ANOVA with Bonferroni *post-hoc* test).

Heart weight-to-tibia length (HW/TL) ratio was significantly elevated in WT mice exposed to chronic hypoxia; however, it was not significantly different between normoxia and hypoxia in *Agat^−/−^* mice supplemented with creatine (Figure 4A). Of note, there was a significant increase in Fulton index in both, *Agat^−/−^* mice supplemented with creatine (0.43 ± 0.04 vs. 0.27 ± 0.05, *p* < 0.001) and unsupplemented WT mice after 4 weeks of hypoxia (0.43 ± 0.06 vs. 0.29 ± 0.04, *p* < 0.001; Figure 4B) as compared with normoxic littermates. Hypoxia did not change tibia length or the sum of LV and septum (Supplementary Figure 2).

**Figure 5:**
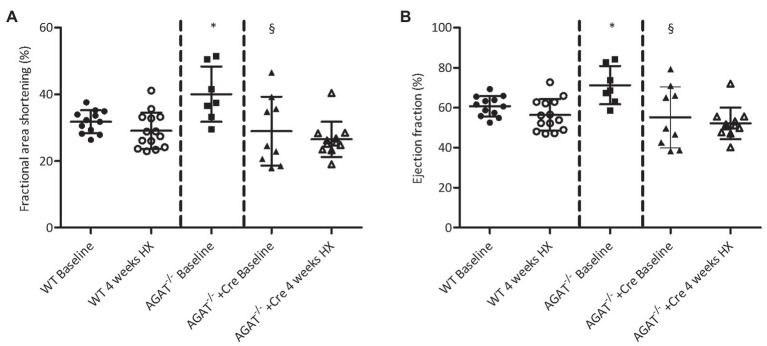
Echocardiography of *Agat^−/−^* mice and WT littermates. **(A,B)** B-mode images were obtained in a parasternal short- and long-axis view, and dimensions of the left ventricle were measured in a short-axis view in diastole and systole from *Agat^−/−^* mice supplemented with 1% creatine and WT littermates (WT) at baseline and after 4 weeks of hypoxia (HX). Mean values ± SD are presented. **(A)** Fractional area shortening. **(B)** Ejection fraction. *Agat*, arginine:glycine amidinotransferase; Cre, supplemented with 1% creatine. ^*^p < 0.05 vs. WT baseline, ^§^*p* < 0.05 vs. *Agat^−/−^* baseline (ANOVA with Bonferroni *post-hoc* test).

Echocardiography revealed higher FAS and ejection fraction in *Agat^−/−^* mice as compared with WT littermates and *Agat^−/−^* mice supplemented with creatine (Figures 5A,B). Hypoxia did not change FAS and ejection fraction in WT mice or AGAT^−/−^ mice supplemented with creatine.

## Discussion

The main findings of our study are threefold: (1) Chronic hypoxia downregulates *Agat* expression and reduces hArg plasma concentrations. (2) *Agat*-deficient mice are not viable in hypoxia. (3) Supplementation with creatine rescues the lethal phenotype and cardiac hypertrophy.

The enzyme AGAT catalyzes the formation of guanidinoacetate from L-glycine and L-arginine; guanidinoacetate is subsequently methylated to creatine by GAMT activity (Kan et al., 2004; Barsunova et al., 2020). AGAT is involved not only in creatine synthesis but also in the formation of hArg from L-arginine and L-lysine (Kayacelebi et al., 2015). Relative mRNA expression of *Agat* and *Gamt* was highest in kidney and liver, respectively (Figure 3). Under hypoxia, *Agat* and *Gamt* were found downregulated in kidney and liver, respectively. Downregulation of *Agat* mRNA was paralleled by decreased AGAT protein detected in the kidney. Moreover, we found impaired hArg in the circulation of hypoxic WT mice. We thus conclude that renal AGAT is essential for hArg synthesis and whole body supply *via* the circulation.

hArg is a non-essential amino acid that differs from arginine by an additional methylene group in the carboxylic acid chain (Bell, 1962). Both L-arginine and hArg serve as substrates for NOS, with a 5- to 15-fold higher catalytic efficiency for L-arginine (Moali et al., 1998). Furthermore, hArg has been shown to be a weak inhibitor of the L-arginine-degrading enzyme arginase (Hrabák et al., 1994). In various clinical studies, low hArg concentrations have been associated with an increased risk of cardiovascular disease and mortality, as recently reviewed by our group (Atzler et al., 2015). Moreover, hArg plasma concentrations in treatment-naive PH patients are low and indicate a risk for mortality (Atzler et al., 2016). In line with these observations in PH patients, chronic hypoxia decreased hArg plasma concentration as well as *Agat* mRNA expression in mice. While this decrease in AGAT expression and function might be maladaptive, the absence of AGAT under hypoxia was deleterious. *Agat^−/−^* mice were not viable in hypoxia for more than 2 days. This phenotype was reversed by supplementation with 1% creatine from weaning and enabled *Agat^−/−^* mice to survive under hypoxic conditions for the complete study period of 4 weeks.

In neuronal and in muscle cells, creatine is phosphorylated to phosphocreatine by creatine kinase, thus serving as a quickly available energy buffer (Lygate et al., 2013; Nabuurs et al., 2013). Adenosine monophosphate-activated protein kinase (AMPK) is a key enzyme regulating energy metabolism; it downregulates energy-consuming metabolic processes in the presence of ATP deficiency. AMPK has been shown to be involved in hypoxic pulmonary vasoconstriction (Evans et al., 2015). In the event of a lack of energy in the cells, AMPK upregulates voltage-dependent potassium channels (Kv5.1), which contributes to cellular repolarization in pulmonary artery smooth muscle cells (Firth et al., 2011). In line with this, we previously observed chronic activation of AMPK in skeletal muscle of AGAT-deficient mice (Choe et al., 2013b). Even though we do not show AMPK activation in tissues of *Agat^−/−^* mice in hypoxia in the current study, we observed a dramatic decrease of blood lactate in *Agat^−/−^* mice as early as 6 h after the initiation of hypoxia. The heart can utilize glucose, lactate, fatty acids, and creatine for maintaining energy supply (Neubauer, 2007; Branovets et al., 2021). The decrease of blood lactate may thus indicate an increased anaerobic energy demand triggered by hypoxia, which cannot be buffered by cellular stores of creatine/phosphocreatine in the heart of *Agat^−/−^* mice as previously shown in hypoxic muscle tissue of these mice (Nabuurs et al., 2013).

Four weeks of hypoxia caused a significant increase of hematocrit as well as cardiac and – more specifically – right ventricular hypertrophy, as assessed by the HW/TL ratio for global cardiac hypertrophy and by the Fulton index for right ventricular hypertrophy. Clearly, the same extent of right ventricular hypertrophy was also found in *Agat^−/−^* mice supplemented with creatine, suggesting that right ventricular hypertrophy is not prevented by creatine but hArg supplementation might have an impact. Global cardiac hypertrophy seemed less pronounced in *Agat^−/−^* mice supplemented with creatine than in unsupplemented WT mice; however, this finding may have been influenced by the slightly reduced total body growth of the *Agat^−/−^* mice supplemented with creatine.

Our study has several limitations. Firstly, *Agat^−/−^* mice were supplemented with creatine from weaning; therefore, we cannot extend our findings to a possible effect of creatine supplementation if initiated after the induction of hypoxia. Secondly, animal welfare prevented us from studying the effects of hArg supplementation on the phenotype of *Agat^−/−^* mice. Supplementation of WT animals with hArg in hypoxia might be an alternative strategy; however, this was beyond the focus of our study. Although hArg has been implicated with heart failure and cardiomyocyte contractility (Faller et al., 2018), we have no definite proof if the PH-like phenotype, i.e., increase in Fulton index, may be caused, at least in part, by lack of hArg.

## Conclusion

We show here that creatine rescues lethality of *Agat^−/−^* mice in hypoxia, showing a pathophysiological significance of creatine-related metabolic pathways. Creatine may therefore be a potentially valuable supplement in individuals having impaired AGAT activity, e.g., caused by genetic missense polymorphisms in the *Agat* gene, during conditions of impaired oxygen availability, e.g., at high altitude. However, creatine supplementation did not affect the PH-like phenotype in hypoxia, suggesting that mechanisms other than creatine-related ones may be involved in its pathogenesis. Further studies in animals and humans are needed to investigate the potentially differential roles of creatine and hArg in PH.

## Data Availability Statement

The raw data supporting the conclusions of this article will be made available by the authors, without undue reservation.

## Ethics Statement

The animal study was reviewed and approved by the Animal Care and Use Committee of the Freie und Hansestadt Hamburg (Approval no. 40/16), BGV, Billestrasse 80, 20539 Hamburg, Germany. Written informed consent was obtained from the owners for the participation of their animals in this study.

## Author Contributions

JH, KC, RB, and ES contributed to the conception and design of the study. KC organized the database. JH, KC, FW, and ES performed the statistical analysis. JH and ES wrote the first draft of the manuscript. KC, AS, JDE, and FW wrote the sections of the manuscript. All authors contributed to manuscript revision, read, and approved the submitted version.

## Conflict of Interest

The authors declare that the research was conducted in the absence of any commercial or financial relationships that could be construed as a potential conflict of interest.

## Publisher’s Note

All claims expressed in this article are solely those of the authors and do not necessarily represent those of their affiliated organizations, or those of the publisher, the editors and the reviewers. Any product that may be evaluated in this article, or claim that may be made by its manufacturer, is not guaranteed or endorsed by the publisher.
