# Supplementary figures and images for: Arginine:Glycine Amidinotransferase Is Essential for Creatine Supply in Mice During Chronic Hypoxia
Source: Front Physiol. 2021 Aug 18;12:703069. doi: 10.3389/fphys.2021.703069 (PMC8416470; doi:10.3389/fphys.2021.703069)

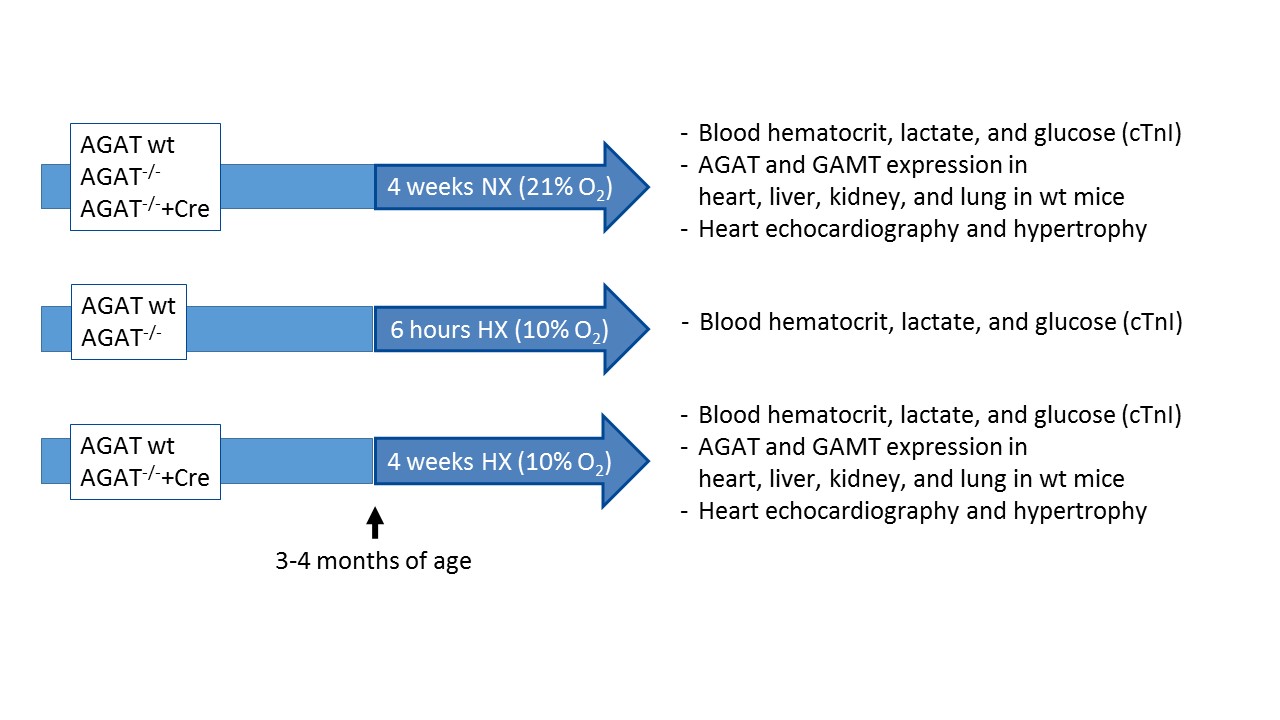

Supplement: Supplementary file 1 [file Image_1.JPEG]

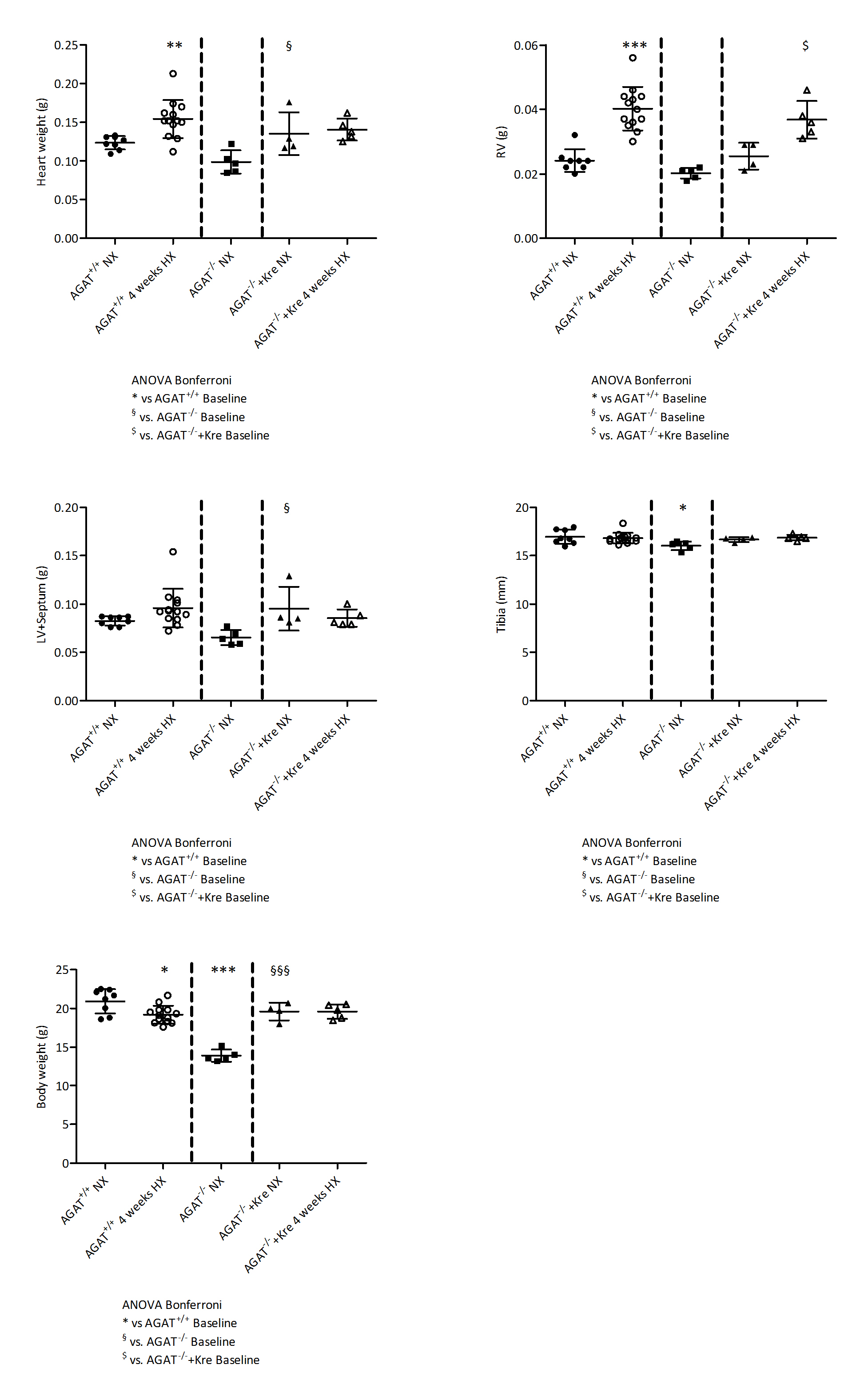

Supplement: Supplementary file 2 [file Image_2.JPEG]
